# Surface Electromyography-Based Motion Analysis of Thigh Muscle Activation During the Modified Star Excursion Balance Test in Novice Recreational Runners with Chronic Ankle Instability: A Preliminary Cross-Sectional Case–Control Study

**DOI:** 10.3390/bioengineering13070846

**Published:** 2026-07-22

**Authors:** Gyu Bin Lee, Jun-Sik Kim, Jin-hwa Lee, Dongyeop Lee

**Affiliations:** 1 Department of Physical Therapy, Sun Moon University, Asan 31460, Republic of Korea; humanwellness99@gmail.com; 2 Chungnam High School, Daejeon 35249, Republic of Korea; gsad1234@hanmail.net; 3 Department of Rehabilitation Medicine, Uijeongbu St. Mary’s Hospital, Uijeongbu 11765, Republic of Korea; aieo7347@gmail.com

**Keywords:** chronic ankle instability, surface electromyography, motion analysis, dynamic postural control, modified Star Excursion Balance Test, neuromuscular control, rectus femoris, recreational runners

## Abstract

Chronic ankle instability (CAI) is associated with impaired sensorimotor function and dynamic postural control; however, reach distance alone may not fully capture task-specific neuromuscular strategies during functional balance tasks. This preliminary cross-sectional case–control study examined modified Star Excursion Balance Test (mSEBT) performance and thigh muscle activation during the mSEBT in novice recreational runners with CAI. Thirty-two novice recreational runners were classified into a CAI group (*n* = 16) or a healthy control group (*n* = 16). Normalized mSEBT reach distances, composite score, weight-bearing lunge test performance, and surface electromyography (sEMG) activity of the rectus femoris, vastus lateralis, vastus medialis, and biceps femoris were analyzed. The anterior reach direction was designated as the primary functional and sEMG task before the formal analyses. No significant between-group differences were observed in functional performance or anterior-task thigh muscle activation. Within the CAI group, anterior reach distance was shorter in the involved limb before adjustment (*p* = 0.014), but the difference did not remain significant after false discovery rate (FDR) correction (q = 0.120). In contrast, rectus femoris (RF) activation during anterior reaching was significantly lower in the involved limb than in the uninvolved limb (*p* = 0.002, q = 0.035) and remained significant after FDR correction. In this preliminary sample, sEMG provided complementary information to mSEBT reach distance by identifying a side-specific difference in RF activation within novice recreational runners with CAI.

## 1. Introduction

Running is a popular activity in modern society that offers numerous health benefits [1], and in recent years, it has continued to gain popularity among people with varying levels of training experience, including novice recreational runners [2]. Despite these benefits, running places repetitive mechanical stress on the lower limbs and requires dynamic postural control during repeated single-limb support. Compared with non-running or less active healthy individuals, runners may exhibit task-specific neuromuscular adaptations related to repeated single-limb support, impact absorption, and forward propulsion, which may influence dynamic postural control [3,4]. Consequently, running carries a risk of musculoskeletal injury; a recent systematic review reported injury incidence and prevalence rates of approximately 40% and 45%, respectively [5]. Among these injuries, ankle and foot injuries are among the most commonly reported lower-limb injuries in runners, and ankle sprains, in particular, are one of the most common sports-related injuries [6,7].

While many people recover from an initial ankle sprain, a significant number may develop chronic ankle instability (CAI), which is characterized by recurrent giving way, a feeling of instability, and reduced function [8,9,10]. CAI cannot be explained solely by ligament laxity or structural instability; it involves sensorimotor impairment, proprioceptive dysfunction, neuromuscular dysregulation, and reduced dynamic postural stability [11,12,13,14]. These deficits may be particularly relevant to runners, who must repeatedly adjust their lower-body alignment and stability when supporting their weight on one leg or changing posture.

In individuals with CAI, dynamic postural control has commonly been assessed using functional reach tasks such as the Star Excursion Balance Test (SEBT) and modified Star Excursion Balance Test (mSEBT) [15,16,17]. The mSEBT is a shortened version of the original eight-direction SEBT and uses the anterior, posteromedial, and posterolateral directions, which have been suggested to retain clinically relevant information on dynamic postural control while reducing redundancy and testing burden [16,17]. The mSEBT has demonstrated good to excellent intra- and inter-rater reliability and is considered a clinically useful functional reach task for identifying dynamic postural-control impairments following lower-extremity injury [16,18].

Among these directions, the anterior reach was selected as the primary functional and surface electromyography (sEMG) task in the present study. This decision was based on evidence that anterior reach asymmetry is associated with the risk of lower-extremity injury [19,20] and that the task requires sagittal-plane control of the stance limb during forward displacement, involving ankle dorsiflexion and movements of the knee and hip [3,4,21,22,23].

Accordingly, the anterior reach direction was selected as the primary task for examining thigh muscle activation in relation to sagittal-plane lower-limb control, not because it was assumed to be more dependent on the thigh muscles than the posteromedial or posterolateral directions. The posteromedial and posterolateral directions were retained for secondary exploratory sEMG analyses. Nevertheless, reach distance represents the final performance outcome of the task, and similar distances may be achieved through different combinations of ankle, knee, hip, and trunk strategies. Therefore, reach distance alone may not fully identify altered muscle recruitment or compensatory postural-control strategies during dynamic balance tasks [24,25,26].

Recent bio-inspired and data-driven engineering studies have emphasized task-specific movement analysis and the integration of biomechanical and neuromuscular data in injury-related assessment [27,28,29]. sEMG provides information about muscle activation during functional movement tasks and may identify neuromuscular changes that are not apparent from reach distance alone. Previous studies have reported altered activation of ankle-related muscles, including the tibialis anterior, peroneus longus, and medial gastrocnemius, and proximal muscles, including the gluteal muscles, rectus femoris, and trunk musculature, in individuals with CAI during balance and rehabilitation tasks [24,25,26,30,31,32]. Muscle activity in these studies was commonly quantified using integrated electromyography (EMG) or normalized amplitude indices, including root mean square (RMS) amplitude relative to maximum voluntary isometric contraction (MVIC) [25,26,31]. However, evidence regarding thigh muscle activation during the mSEBT in novice recreational runners with CAI remains limited. Running requires repeated single-limb support and propulsion, with coordinated knee stabilization and lower-limb control. Thigh muscles, including the rectus femoris (RF), vastus lateralis (VL), vastus medialis (VM), and biceps femoris (BF), may contribute to these functions during dynamic balance tasks [33,34]. Examining these muscles during the mSEBT may therefore provide insight into task-specific neuromuscular control in novice recreational runners with CAI.

The purpose of this study was to compare mSEBT performance and thigh muscle activity during task performance between novice recreational runners with CAI and healthy novice recreational runners. Additionally, we sought to investigate differences between the involved and uninvolved limbs within the CAI group. Based on the rationale outlined above, the anterior reach direction was designated as the primary functional and sEMG task, whereas the posteromedial and posterolateral directions were examined as secondary exploratory sEMG tasks. We hypothesized that novice recreational runners with CAI would show differences in mSEBT performance and thigh muscle activation compared to a healthy control group, and that side-to-side differences would be observed between the involved and uninvolved limbs within the CAI group.

## 2. Materials and Methods

### 2.1. Study Design and Participants

This preliminary cross-sectional case–control study included 32 novice recreational runners who were recruited using convenience sampling from the local community and classified into either the CAI group (*n* = 16) or the healthy control group (*n* = 16). Novice recreational runners were defined as non-competitive runners who had been running regularly for less than two years [35]. The additional running-related eligibility criteria were as follows: (1) aged 18–39 years; (2) an average weekly running distance of 20 km or less; and (3) the self-reported ability to run continuously for 3 km or 20 min. No fixed minimum weekly running distance was applied.

The inclusion criteria for CAI participants and healthy controls were determined in accordance with the recommendations of the International Ankle Consortium [36]. Participants in the CAI group were required to have experienced at least one significant ankle sprain at least 12 months prior to enrollment, with no acute ankle sprain within the previous 3 months. They were also required to meet the following inclusion criteria: (1) a Cumberland Ankle Instability Tool (CAIT) score of ≤24; (2) at least five affirmative responses on the Ankle Instability Instrument (AII), including an affirmative response to item 1; and (3) at least two episodes of giving way within the previous six months.

The healthy control group comprised novice recreational runners who met the same general running-related inclusion criteria as the CAI group. To be eligible for the healthy control group, participants were required to have no history of ankle ligament injury, no perceived ankle instability, and a Cumberland Ankle Instability Tool (CAIT) score of ≥28.

The same exclusion criteria were applied to both groups. These included a musculoskeletal injury within the previous 3 months, a history of lower-extremity surgery within the previous 6 months, a systemic or neurological condition that could affect the study outcomes, and alcohol consumption or medication use within 24 h before testing that could influence physical performance. Eligibility and exclusion criteria were verified before enrollment through a screening assessment conducted by the first author (G.B.L.).

This study was approved by the Institutional Review Board (IRB) at Sun Moon University (approval number: SM-202311-033-2), and written informed consent was obtained from all participants.

### 2.2. Sample Size Consideration

Because this preliminary cross-sectional case–control study was conducted using a convenience sample of novice recreational runners who met the eligibility criteria, an a priori sample size calculation was not performed. To clarify the interpretability of statistically non-significant findings, a sensitivity power analysis was conducted using G*Power version 3.1.9.7 (Heinrich Heine University Düsseldorf, Düsseldorf, Germany) [37]. For between-group comparisons, assuming 16 participants per group, a two-sided alpha level of 0.05, and 80% statistical power, the available sample size was sufficient to detect only large effects of approximately Cohen’s d ≥ 1.02. For within-group side-to-side comparisons in the CAI group, based on 16 paired observations, a two-sided alpha level of 0.05, and 80% statistical power, the sample was sufficient to detect effects of approximately Cohen’s dz ≥ 0.75. Therefore, non-significant findings, particularly those from between-group comparisons, should be interpreted with caution and should not be regarded as evidence of equivalence between the groups.

### 2.3. Experimental Procedures

All testing was conducted in the laboratory of the Department of Physical Therapy at Sun Moon University, Asan, Republic of Korea. Before functional assessment, participant characteristics (age, height, weight, body mass index [BMI], dominant limb, and running history) and ankle-sprain history were recorded. Limb length was measured bilaterally from the anterior superior iliac spine to the distal point of the medial malleolus with the participant supine. Weight-bearing ankle dorsiflexion was assessed bilaterally using the Weight-Bearing Lunge Test (WBLT).

In the CAI group, the involved limb was defined as the limb with chronic ankle instability. Because healthy controls had no clinically involved limb, a matched reference limb was assigned in the healthy control group according to the lateral distribution of the involved limbs in the CAI group. Specifically, because the involved limbs in the CAI group included 10 right limbs and 6 left limbs, 10 right and 6 left limbs were assigned as matched reference limbs in the healthy control group. This was a distribution-matching procedure rather than a randomization procedure, and it was used only to define the limb for between-group comparisons. It did not indicate pathological involvement in the healthy control group.

sEMG was recorded during the mSEBT. Before the mSEBT, participants performed a 5 min standardized warm-up on a stationary bicycle at a comfortable self-selected intensity. No static stretching or high-intensity exercise was included before testing [38]. All assessments were conducted by the first author (G.B.L.), a physical therapist with extensive experience in musculoskeletal and functional testing relevant to the study. The examiner was not blinded to group status. The second author holds a Ph.D. in Physical Education and has research experience in sports biomechanics and electromyographic movement analysis. He assisted with participant recruitment, scheduling, testing coordination, data-collection logistics, and preparation of the testing environment; however, the primary clinical and sEMG procedures, including the WBLT, mSEBT, MVIC procedures, and electrode placement, were performed by the first author.

### 2.4. Surface Electromyography Measurement

**Figure 1:**
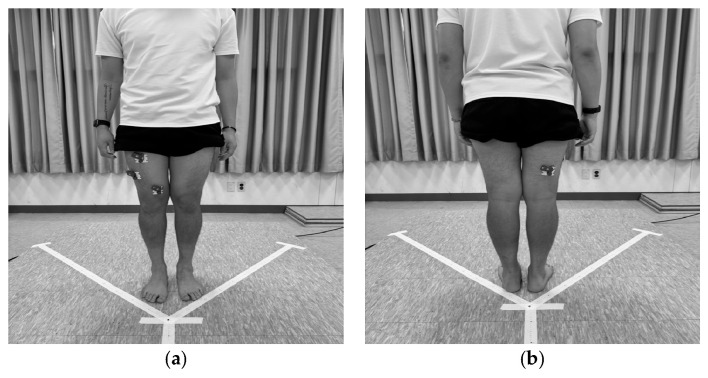
Surface electromyography electrode placement for the thigh muscles: (**a**) anterior view showing the rectus femoris, vastus lateralis, and vastus medialis electrode positions; (**b**) posterior view showing the biceps femoris electrode position.

Muscle activity was recorded from the rectus femoris (RF), vastus medialis (VM), vastus lateralis (VL), and biceps femoris (BF) using a wireless surface EMG system (FREEEMG 1000, BTS Bioengineering S.p.A., Garbagnate Milanese, Italy). Bipolar surface electrodes were placed parallel to the muscle fibers with a 20 mm interelectrode distance in accordance with the SENIAM recommendations [39] (Figure 1).

Before electrode placement, the skin was cleaned with alcohol to reduce skin–electrode impedance, and hair was removed when necessary. Before data processing, the EMG signals recorded during the MVIC and task trials were visually inspected for obvious noise or signal loss.

MVIC data for knee extension and flexion were collected to normalize the EMG signals. For the knee extension MVIC, participants sat on a treatment table with the hip and knee flexed to approximately 90°. A non-elastic fixation strap was secured around the distal lower leg, immediately proximal to the ankle, to provide resistance against knee extension. For the knee flexion MVIC, participants lay prone on the treatment table with the knee flexed to approximately 90°, and the fixation strap was placed around the distal lower leg to resist knee flexion. Standing or task-specific MVIC positions were not used in this study because they may impose additional postural demands during maximal contraction and increase the likelihood of compensatory movements involving the trunk or hip.

During each MVIC trial, participants were instructed to gradually increase their effort to the maximum level following the start sign and maintain the contraction for 5 s. After one familiarization trial, three MVIC trials were performed for each movement, with 30 s of rest between trials. Standardized verbal encouragement was provided by the examiner during each MVIC trial. For each MVIC trial, the RMS amplitude over the middle 3 s of the 5 s contraction was calculated. The mean value from the three trials was then used as the normalization reference for each muscle.

Raw EMG signals were sampled at 1000 Hz and processed using EMG Analyzer software (version 2.9.40.0; BTS Bioengineering S.p.A., Garbagnate Milanese, Italy). The signals were band-pass filtered between 20 and 450 Hz using a fourth-order Butterworth filter, full-wave rectified, and processed using an RMS algorithm with a 50 ms moving window. The mean RMS amplitude recorded for each muscle during the mSEBT was normalized to the corresponding MVIC value and expressed as a percentage of MVIC (%MVIC). Each analysis interval extended from the instant the reaching limb left the ground until it returned to the starting position. The mean value obtained from three successful trials in each reach direction was used for the final analysis.

### 2.5. Modified Star Excursion Balance Test

**Figure 2:**
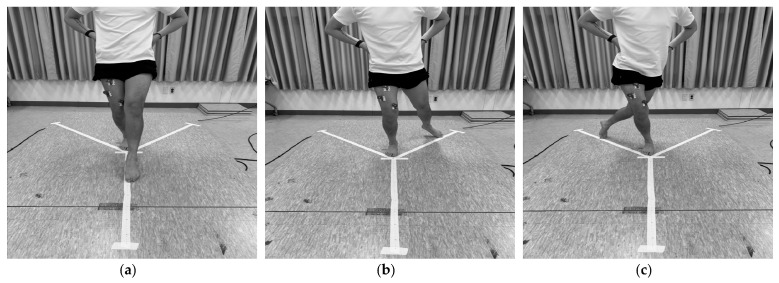
Modified Star Excursion Balance Test setup showing the three reach directions: (**a**) anterior, (**b**) posteromedial, and (**c**) posterolateral.

Dynamic balance was assessed using the mSEBT, which has demonstrated good to excellent inter-rater and intra-rater reliability [18]. Participants stood on one foot at the center of the test grid and reached with the contralateral limb in the anterior (ANT), posteromedial (PM), and posterolateral (PL) directions. One anterior line and two posterior lines oriented at 135° to the anterior line were marked on the floor (Figure 2).

The mSEBT was administered in accordance with previous studies [16,40]. Participants performed the test barefoot with their hands on their hips. An attempt was considered invalid if the participant failed to maintain single-limb stance, lifted or moved the stance foot, placed substantial weight through the reaching foot, or could not return the reaching foot to the starting position under control. Before recorded testing, participants performed six practice trials in each direction as a conservative familiarization procedure. Previous SEBT learning-effect studies have reported that reach distances generally stabilize within approximately four practice trials, and mSEBT reproducibility studies have used practice trials before recorded testing [18,41,42]. After a 5 min rest period, participants completed three recorded trials in each direction. Reach distance was measured in centimeters, and the mean of three successful trials was used. Reach distances were normalized to limb length (normalized reach distance [%] = reach distance/limb length × 100), and the composite score was calculated as the mean of the normalized ANT, PM, and PL reach distances.

Lower-extremity sEMG data were recorded concurrently during the mSEBT. To ensure consistency in movement speed, all participants practiced maintaining a steady pace in time with an 80-bpm metronome before testing. Before the formal statistical analyses, the anterior reach direction was designated as the primary functional and sEMG task. The posteromedial and posterolateral reach directions were analyzed as secondary exploratory sEMG tasks, and the corresponding results are presented in the Supplementary Materials.

### 2.6. Weight-Bearing Lunge Test

Ankle dorsiflexion range of motion was measured using the WBLT. Participants kept the heel in contact with the floor and advanced the knee toward the wall. The maximum toe-to-wall distance achieved while maintaining heel contact was measured in centimeters [43]; a greater distance indicated greater weight-bearing ankle dorsiflexion [44]. Each participant performed three practice trials followed by three test trials, and the mean of the three test trials was used for analysis. The test was performed bilaterally. Previous studies have demonstrated high intra-rater reliability for the WBLT [43,45].

### 2.7. Statistical Analysis

Statistical analyses were performed using IBM SPSS Statistics version 25.0 (IBM Corp., Armonk, NY, USA), and Benjamini–Hochberg false discovery rate (FDR)-adjusted *p*-values were calculated separately using R version 4.5.2 (R Foundation for Statistical Computing, Vienna, Austria). The Shapiro–Wilk test was used to assess the normality of continuous variables, and descriptive statistics are presented as mean ± standard deviation. Participant characteristics were compared between groups using independent *t*-tests or Mann–Whitney U tests for continuous variables, depending on data normality. Categorical variables were compared using the chi-square test or Fisher’s exact test, depending on the expected cell counts.

For between-group analyses, functional performance and anterior-task sEMG variables were compared between the involved limb of the CAI group and the matched reference limb of the healthy control group using Welch’s independent *t*-tests. Levene’s test results for these comparisons are provided in Supplementary Table S2. Hedges’ g was calculated as the effect size. Within the CAI group, side-to-side differences between the involved and uninvolved limbs were analyzed using paired *t*-tests, and Cohen’s dz was calculated as the effect size. Wilcoxon signed-rank tests were additionally conducted as sensitivity analyses for variables with non-normally distributed paired differences, and the conclusions remained unchanged.

The Benjamini–Hochberg FDR procedure was applied to correct for multiple comparisons. For the between-group analyses, FDR correction was applied across nine variables: the WBLT, three mSEBT reach directions, the composite score, and four anterior-task sEMG variables. For the within-CAI side-to-side analyses, FDR correction was applied across 17 predefined variables, including the WBLT, mSEBT outcomes, and anterior, posteromedial, and posterolateral sEMG variables. Adjusted *p*-values are reported as FDR q-values. Predefined pairwise comparisons were used because only the CAI group had clinically defined involved and uninvolved limbs; a Group × Side interaction based on an assigned healthy reference limb would address a different and less clinically interpretable estimand. For the predefined outcome families, statistical significance was determined using an FDR-adjusted q-value < 0.05; unadjusted *p*-values are reported for transparency.

## 3. Results

### 3.1. Participant Characteristics

A total of 32 novice recreational runners were included in the final analysis, with 16 classified in the CAI group and 16 in the healthy control group. The groups did not differ significantly in sex distribution, age, height, weight, BMI, or reference-limb length (all *p* > 0.05). The CAI group had significantly lower CAIT scores and higher AII scores than the healthy control group (both *p* < 0.001). Participant characteristics and self-reported ankle function are presented in Table 1.

### 3.2. Between-Group Comparison of Functional Performance

We compared functional performance between the involved limb in the CAI group and the matched reference limb in the healthy control group. No significant between-group differences were observed in WBLT, mSEBT anterior, posteromedial, or posterolateral reach distance, or composite score after FDR correction (Table 2). There was no significant difference in the mSEBT anterior reach distance between the involved limb in the CAI group and the matched reference limb in the healthy control group (69.91 ± 6.10% vs. 68.64 ± 7.32%, *p* = 0.599, Hedges’ g = 0.183). Similarly, no significant between-group differences were observed in posteromedial reach distance (113.97 ± 11.58% vs. 111.38 ± 11.81%, *p* = 0.537, Hedges’ g = 0.215), posterolateral reach distance (107.39 ± 14.08% vs. 103.25 ± 13.43%, *p* = 0.402, Hedges’ g = 0.293), or composite score (97.09 ± 9.61% vs. 94.43 ± 9.66%, *p* = 0.441, Hedges’ g = 0.269).

### 3.3. Between-Group Comparison of Thigh Muscle Activation During the Anterior Task

We compared sEMG activity during the anterior reach task between the involved limb of the CAI group and the matched reference limb of the healthy control group. No significant between-group differences were observed in RF, VL, VM, or BF activation during the anterior reach task after FDR correction (Table 3). In the involved limb, RF activation was lower than in the matched reference limb of the healthy control group, but this difference was not statistically significant (15.85 ± 7.57%MVIC vs. 19.65 ± 10.38%MVIC, *p* = 0.246, Hedges’ g = −0.408). Similarly, no significant between-group differences were observed in VL activation (41.26 ± 19.78%MVIC vs. 42.73 ± 11.70%MVIC, *p* = 0.800, Hedges’ g = −0.088), VM activation (47.10 ± 27.87%MVIC vs. 42.88 ± 14.98%MVIC, *p* = 0.599, Hedges’ g = 0.184), or BF activation (11.46 ± 5.90%MVIC vs. 11.80 ± 5.04%MVIC, *p* = 0.864, Hedges’ g = −0.060). The results of the secondary analysis of the posteromedial and posterolateral sEMG variables are presented in Supplementary Table S1.

### 3.4. Side-to-Side Comparison Within the CAI Group

Within the CAI group, a side-to-side comparison was performed between the involved and uninvolved limbs (Table 4). The mSEBT anterior reach distance was shorter for the involved limb than for the uninvolved limb (69.91 ± 6.10% vs. 73.19 ± 4.56%, *p* = 0.014, Cohen’s dz = −0.694). However, this difference was not statistically significant after FDR correction (q = 0.120). The mSEBT composite score did not show a significant difference between the involved and uninvolved limbs (97.09 ± 9.61% vs. 98.04 ± 7.17%, *p* = 0.353, q = 0.546, Cohen’s dz = −0.239).

**Figure 3:**
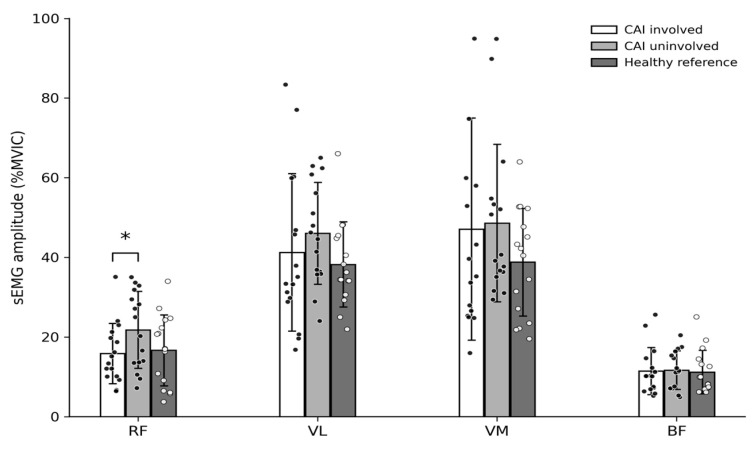
Normalized anterior-task surface electromyography (sEMG) amplitudes (%MVIC) of the rectus femoris (RF), vastus lateralis (VL), vastus medialis (VM), and biceps femoris (BF) during the anterior reach task of the modified Star Excursion Balance Test (mSEBT). Bars represent mean values, error bars indicate standard deviations, and dots represent individual participants. * Significant side-to-side difference within the CAI group after FDR correction. CAI, chronic ankle instability; MVIC, maximum voluntary isometric contraction; FDR, false discovery rate; Healthy reference, healthy matched reference limb.

In terms of sEMG activity during the anterior reach task, RF activation was significantly lower in the involved limb than in the uninvolved limb (15.85 ± 7.57%MVIC vs. 21.80 ± 9.65%MVIC, *p* = 0.002, q = 0.035, Cohen’s dz = −0.931). No significant side-to-side differences were observed in VL, VM, or BF activation during the anterior reach task. The normalized anterior-task sEMG amplitudes of RF, VL, VM, and BF are visually summarized in Figure 3. The complete results for all 17 predefined within-CAI side-to-side comparisons are presented in Supplementary Table S3.

**Table 4:** Side-to-side comparison of functional performance and anterior-task sEMG activity within the CAI group.

Variable	Involved Limb	Uninvolved Limb	*p*-Value	FDR q	Cohen’s dz
WBLT (cm)	12.06 ± 2.90	12.75 ± 1.82	0.217	0.411	−0.322
mSEBT anterior	69.91 ± 6.10	73.19 ± 4.56	0.014	0.120	−0.694
mSEBT composite	97.09 ± 9.61	98.04 ± 7.17	0.353	0.546	−0.239
Anterior RF	15.85 ± 7.57	21.80 ± 9.65	0.002	0.035 *	−0.931
Anterior VL	41.26 ± 19.78	46.05 ± 12.78	0.135	0.327	−0.395
Anterior VM	47.10 ± 27.87	48.61 ± 19.78	0.700	0.866	−0.098
Anterior BF	11.46 ± 5.90	11.68 ± 4.84	0.872	0.927	−0.041

Values are presented as mean ± standard deviation. sEMG values are expressed as %MVIC. CAI, chronic ankle instability; WBLT, weight-bearing lunge test; mSEBT, modified Star Excursion Balance Test; RF, rectus femoris; VL, vastus lateralis; VM, vastus medialis; BF, biceps femoris; FDR, false discovery rate. FDR q-values were calculated using the Benjamini–Hochberg procedure across 17 predefined within-CAI side-to-side comparisons, including WBLT, mSEBT reach outcomes, and anterior, posteromedial, and posterolateral sEMG variables. Cohen’s dz was calculated as the involved limb minus the uninvolved limb; negative values indicate lower values in the involved limb. * Statistically significant after FDR correction (q < 0.05).

## 4. Discussion

The aim of this preliminary cross-sectional case–control study was to compare mSEBT performance and thigh muscle activation during the mSEBT between novice recreational runners with CAI and healthy novice recreational runners. Side-to-side differences between the involved and uninvolved limbs were also examined within the CAI group. The main findings were as follows. First, no significant between-group differences were observed in WBLT performance, mSEBT performance, or thigh muscle activation during the anterior reach task between the involved limb of the CAI group and the matched reference limb of the healthy control group. The matched reference limb in the healthy control group was used only as an analytic reference for the between-group comparison. Because this limb had no injury history or clinical involvement, the between-group comparison should not be interpreted as a true biological involved-limb comparison. Second, within the CAI group, anterior reach performance was lower in the involved limb than in the uninvolved limb at the unadjusted level; however, this difference was no longer statistically significant after FDR correction. Third, RF activation during the anterior reach task was significantly lower in the involved limb than in the uninvolved limb within the CAI group, and this difference remained significant after FDR correction. These findings suggest that side-specific neuromuscular differences may be present during anterior reaching in novice recreational runners with CAI. Nevertheless, the results should be interpreted as preliminary and hypothesis-generating.

No significant between-group differences in mSEBT performance were observed, and the findings did not support the initial hypothesis of impaired reach performance in the CAI group. Previous studies have reported shorter mSEBT reach distances in individuals with CAI than in healthy controls or ankle-sprain copers [15,25,46,47,48]. Reach performance can be influenced by muscle strength, flexibility, ankle dorsiflexion, physical activity, and neuromuscular control [16,49,50]. Repeated exposure to single-limb support and forward propulsion during running may have contributed to preserved performance; however, running volume, training frequency, and biomechanics were not quantified, so this explanation remains speculative [3,51]. Similar reach distances may also be achieved through different motor strategies.

Because the side-to-side difference in anterior reach distance did not remain significant after FDR correction, it should be interpreted only as a direction-specific tendency, and any biomechanical explanation is speculative. Anterior reach performance may be influenced by sagittal-plane control, ankle dorsiflexion, and lower-limb alignment [22,52], and ankle dorsiflexion has been associated with anterior reach performance [53,54,55,56]. However, no significant side-to-side difference in WBLT performance was observed. The anterior reach result therefore does not provide robust evidence of impaired performance in the involved limb and requires confirmation in larger studies with kinematic analysis.

A noteworthy preliminary finding was lower RF activation in the involved limb than in the uninvolved limb during anterior reach after FDR correction. Because trunk, pelvic, hip, and knee kinematics were not measured, the movement strategy underlying this finding cannot be determined. Previous studies have linked anterior reach performance to knee and hip flexion strategies [23] and have reported trunk rotation and increased hip flexion during the SEBT in individuals with CAI [21,57]; however, these mechanisms were not measured in the present study. The lower RF activation should therefore not be interpreted as direct evidence of impaired knee stability or reduced knee-extension capacity. The absolute difference was approximately 6%MVIC, but no minimal clinically important difference or functional threshold has been established for RF activation during the mSEBT in novice recreational runners with CAI. Its clinical significance is therefore unknown, and the result should be regarded as a preliminary physiological observation. Future studies should combine sEMG with three-dimensional kinematic and kinetic analyses.

Secondary exploratory analyses of the posteromedial and posterolateral directions showed no significant between-group differences in RF, VL, VM, or BF activation. Group-level differences in thigh muscle activation were therefore not consistently observed across mSEBT directions in this preliminary sample. These secondary findings should be interpreted descriptively and support further direction-specific studies combining sEMG with kinematic and kinetic analyses.

From a clinical perspective, incorporating sEMG into dynamic balance tasks may be considered an exploratory research approach for examining task-specific neuromuscular strategies in runners with CAI. The significant result was limited to one within-CAI RF comparison, and no between-group differences in thigh muscle activation were observed; therefore, the findings do not support routine clinical sEMG assessment.

This study has several limitations. First, the sample was small, no a priori sample size calculation was performed, and the sensitivity analysis indicated that only relatively large effects were detectable; non-significant between-group findings should not be interpreted as evidence of equivalence. Second, the findings may not generalize beyond novice recreational runners. Running exposure and task familiarity were not quantified and may have attenuated between-group differences. Third, the healthy matched reference limb was an analytic reference rather than a true biological counterpart to the CAI-involved limb. Because bilateral symmetry was not analyzed in the healthy group, the within-CAI RF difference cannot be attributed uniquely to CAI. Fourth, the study was not preregistered; therefore, the designation of the anterior task and the direction-specific RF result should be considered preliminary and hypothesis-generating. Fifth, kinematic and kinetic data were not collected, and only selected thigh muscles were examined; activation of the ankle, hip, and trunk musculature was not recorded. Sixth, EMG amplitudes were normalized to seated or prone MVICs, whereas the mSEBT was performed during dynamic standing. This limitation may be particularly relevant to the biarticular RF, and the %MVIC values should be interpreted cautiously. Seventh, study-specific intra-rater reliability was not calculated, and all measurements were performed by a single non-blinded physical therapist; inter-rater reliability could not be estimated and assessor bias cannot be excluded. Associations between CAIT or AII scores and sEMG activity were not examined. Finally, the cross-sectional design precludes causal inference. Larger preregistered studies integrating sEMG with kinematic and kinetic analyses are needed.

## 5. Conclusions

In this preliminary cross-sectional case–control study, novice recreational runners with chronic ankle instability did not differ significantly from healthy controls in mSEBT performance or thigh muscle activation during the anterior reach task. However, within the CAI group, RF activation during anterior reaching was significantly lower in the involved limb than in the uninvolved limb, and this difference remained significant after FDR correction. These findings suggest that side-specific neuromuscular differences may be present during anterior reaching even though no statistically significant difference in functional reach performance was detected. Because of the small sample size and the absence of kinematic and kinetic analyses, these results should be interpreted as preliminary and hypothesis-generating.

## Figures and Tables

**Table 1:** Participant characteristics and self-reported ankle function.

Variable	CAI Group (*n* = 16)	Healthy Group (*n* = 16)	*p*-Value
Sex (Male/Female)	6/10	9/7	0.480
Age (years)	30.38 ± 3.69	29.00 ± 3.06	0.260
Height (cm)	165.23 ± 6.55	168.88 ± 8.33	0.179
Weight (kg)	62.61 ± 6.82	65.93 ± 9.74	0.274
BMI (kg/m^2^)	22.88 ± 1.21	22.97 ± 1.57	0.861
Dominant limb (right/left)	16/0	16/0	-
Involved/matched reference limb laterality (right/left)	10/6	10/6	-
Involved/matched reference limb length (cm)	81.57 ± 4.18	83.64 ± 4.73	0.187
CAIT score	18.63 ± 5.33	29.31 ± 1.08	<0.001
AII score	6.50 ± 1.46	0.25 ± 0.45	<0.001

Values are presented as mean ± standard deviation or number of participants. No statistical comparison was performed for dominant-limb distribution because all participants were right-limb dominant. The laterality distribution of the healthy matched reference limbs was fixed by the study design to match that of the CAI involved limbs. CAI, chronic ankle instability; BMI, body mass index; CAIT, Cumberland Ankle Instability Tool; AII, Ankle Instability Instrument.

**Table 2:** Between-group comparison of functional performance between the CAI-involved limb and the healthy matched reference limb.

Variable	CAI Involved Limb	Healthy Matched Reference Limb	*p*-Value	FDR q	Hedges’ g
WBLT (cm)	12.06 ± 2.90	11.72 ± 2.78	0.735	0.864	0.118
mSEBT anterior (%)	69.91 ± 6.10	68.64 ± 7.32	0.599	0.864	0.183
mSEBT posteromedial	113.97 ± 11.58	111.38 ± 11.81	0.537	0.864	0.215
mSEBT posterolateral	107.39 ± 14.08	103.25 ± 13.43	0.402	0.864	0.293
mSEBT composite	97.09 ± 9.61	94.43 ± 9.66	0.441	0.864	0.269

Values are presented as mean ± standard deviation. CAI, chronic ankle instability; WBLT, weight-bearing lunge test; mSEBT, modified Star Excursion Balance Test. mSEBT reach distances are normalized to limb length and expressed as percentages. Hedges’ g was calculated as the CAI involved limb minus the healthy matched reference limb; negative values indicate lower values in the CAI group. FDR q-values were calculated using the Benjamini–Hochberg procedure across the nine between-group variables.

**Table 3:** Between-group comparison of anterior-task sEMG activity between the CAI-involved limb and the healthy matched reference limb.

Muscle	CAI Involved Limb	Healthy Matched Reference Limb	*p*-Value	FDR q	Hedges’ g
RF	15.85 ± 7.57	19.65 ± 10.38	0.246	0.864	−0.408
VL	41.26 ± 19.78	42.73 ± 11.70	0.800	0.864	−0.088
VM	47.10 ± 27.87	42.88 ± 14.98	0.599	0.864	0.184
BF	11.46 ± 5.90	11.80 ± 5.04	0.864	0.864	−0.060

Values are presented as mean ± standard deviation. sEMG values are expressed as %MVIC. CAI, chronic ankle instability; RF, rectus femoris; VL, vastus lateralis; VM, vastus medialis; BF, biceps femoris; MVIC, maximum voluntary isometric contraction. FDR q-values were calculated using the Benjamini–Hochberg procedure across the nine between-group variables. Hedges’ g was calculated as the CAI involved limb minus the healthy matched reference limb; negative values indicate lower activation in the CAI group.

## Data Availability

The data presented in this study are available on request from the corresponding author due to privacy and ethical restrictions related to participant confidentiality and the terms of IRB approval.

## Supplementary Materials

The following supporting information can be downloaded at: https://www.mdpi.com/article/10.3390/bioengineering13070846/s1; Table S1: Between-group comparison of sEMG activity during the posteromedial and posterolateral tasks between the CAI-involved limb and the healthy matched reference limb; Table S2: Levene’s test results for the nine between-group comparisons; Table S3: Complete results for the 17 predefined within-CAI side-to-side comparisons included in the Benjamini–Hochberg false discovery rate correction.
