# Supplementary material for: Surface Electromyography-Based Motion Analysis of Thigh Muscle Activation During the Modified Star Excursion Balance Test in Novice Recreational Runners with Chronic Ankle Instability: A Preliminary Cross-Sectional Case–Control Study
Source: Bioengineering (Basel). 2026 Jul 22;13(7):846. doi: 10.3390/bioengineering13070846 (PMC13405922; doi:10.3390/bioengineering13070846)
Supplement: Supplementary file 1 [file bioengineering-13-00846-s001.zip › bioengineering-4398442-supplementary.pdf]

bioengineering MDPI

Supplementary Materials: The following supporting information can be downloaded at: https://www.mdpi.com/article/10.3390/bioengineering13070846/s1, Table S1: Between-group comparison of sEMG activity during the posteromedial and posterolateral tasks between the CAI-involved limb and the healthy matched reference limb; Table S2: Levene's test results for the nine between-group comparisons; Table S3: Complete results for the 17 predefined within-CAI side-to-side comparisons included in the Benjamini–Hochberg false discovery rate correction.

Table S1. Between-group comparison of sEMG activity during the posteromedial and posterolateral tasks between the CAI involved limb and the healthy matched reference limb.

Variable	CAI involved limb	Healthy matched reference limb	Mean difference	p-value	Hedges' g
Posteromedial RF	31.50 ± 16.07	38.17 ± 21.63	-6.67	0.331	-0.341
Posteromedial VL	49.64 ± 20.14	48.20 ± 15.51	1.44	0.823	0.078
Posteromedial VM	57.15 ± 32.50	47.29 ± 14.78	9.86	0.282	0.380
Posteromedial BF	10.34 ± 4.09	10.37 ± 3.91	-0.03	0.981	-0.008
Posterolateral RF	25.75 ± 12.55	29.16 ± 17.00	-3.41	0.524	-0.222
Posterolateral VL	39.27 ± 16.00	39.02 ± 10.32	0.25	0.959	0.018
Posterolateral VM	46.67 ± 27.61	40.86 ± 10.83	5.81	0.442	0.270
Posterolateral BF	12.68 ± 5.83	13.89 ± 5.29	-1.21	0.543	-0.212

Values are presented as mean ± standard deviation and expressed as %MVIC. Mean differences and Hedges' g were calculated as the CAI involved limb minus the healthy matched reference limb. Negative values indicate lower activation in the CAI group. The p-values are unadjusted and are presented for descriptive exploratory purposes. CAI, chronic ankle instability; sEMG, surface electromyography; MVIC, maximum voluntary isometric contraction; RF, rectus femoris; VL, vastus lateralis; VM, vastus medialis; BF, biceps femoris.

Table S2. Levene's test results for the nine between-group comparisons.

Variable	Levene's test p-value
WBLT	0.780
mSEBT anterior	0.792
mSEBT posteromedial	0.828
mSEBT posterolateral	0.723
mSEBT composite	0.887
Anterior RF	0.170
Anterior VL	0.054
Anterior VM	0.050
Anterior BF	0.786

Levene's test was performed to describe homogeneity of variance for the between-group comparisons. Welch's independent t-tests were used consistently for all between-group analyses regardless of Levene's test results.

Table S3. Complete results for the 17 predefined within-CAI side-to-side comparisons included in the Benjamini–Hochberg false discovery rate correction.

Variable	Involved limb	Uninvolved limb	p-value	FDR q	Cohen's dz
WBLT	12.06 ± 2.90	12.75 ± 1.82	0.217	0.411	−0.322
mSEBT anterior	69.91 ± 6.10	73.19 ± 4.56	0.014	0.120	−0.694
mSEBT posteromedial	113.97 ± 11.58	113.92 ± 9.42	0.977	0.977	0.007
mSEBT posterolateral	107.39 ± 14.08	107.02 ± 11.20	0.782	0.886	0.071
mSEBT composite	97.09 ± 9.61	98.04 ± 7.17	0.353	0.546	−0.239
**Anterior RF**	15.85 ± 7.57	21.80 ± 9.65	**0.002**	**0.035***	−0.931

Anterior VL	41.26 ± 19.78	46.05 ± 12.78	0.135	0.327	−0.395
Anterior VM	47.10 ± 27.87	48.61 ± 19.78	0.700	0.866	−0.098
Anterior BF	11.46 ± 5.90	11.68 ± 4.84	0.872	0.927	−0.041
Posteromedial RF	31.50 ± 16.07	38.00 ± 14.73	0.044	0.250	−0.550
Posteromedial VL	49.64 ± 20.14	55.30 ± 13.98	0.118	0.327	−0.415
Posteromedial VM	57.15 ± 32.50	55.43 ± 20.23	0.713	0.866	0.094
Posteromedial BF	10.34 ± 4.09	12.53 ± 7.25	0.200	0.411	−0.335
Posterolateral RF	25.75 ± 12.55	28.37 ± 16.85	0.481	0.681	−0.181
Posterolateral VL	39.27 ± 16.00	44.53 ± 18.06	0.060	0.256	−0.508
Posterolateral VM	46.67 ± 27.61	43.46 ± 16.57	0.325	0.546	0.255
Posterolateral BF	12.68 ± 5.83	15.37 ± 8.26	0.127	0.327	−0.404

Values are presented as mean ± standard deviation. WBLT values are expressed in centimeters, mSEBT values as percentages of limb length, and sEMG values as %MVIC. p-values were obtained using two-sided paired t-tests. Cohen's dz was calculated as the involved limb minus the uninvolved limb; negative values indicate lower values in the involved limb. FDR q-values were calculated across all 17 comparisons using the Benjamini–Hochberg procedure. Wilcoxon sensitivity analyses for non-normal paired differences did not alter the conclusions. * $q < 0.05$ after FDR correction.

https://doi.org/10.3390/bioengineering13070846
